# The Poor Man's Home.—XVII

**Published:** 1897-12-11

**Authors:** 


### Modern Sociology.

### THE POOR MAN'S HOME.—XVII.

#### Housing in America.

In no country in the world has the experiment of housing the poor had a better chance of success than in America, for America is the land of experiment. We propose to describe an example of housing on a commercial, semi-philanthropic, and a purely philanthropic basis.

For the first, we select for description the Riverside Buildings of the Improved Dwellings Company, of Brooklyn, New York. The older buildings of the company pay as much as 10 per cent.; but the ones we propose to speak of have been less of a success financially. They pay only from 5 to 6½ per cent.—no despicable return from an English point of view—but they are more modern in style, and have various conveniences which the others do not possess; so that they are, therefore, more suitable for our purpose. The Riverside Buildings cover three sides of a quadrangle. Of the space thus enclosed, only half is covered with buildings. Of the remainder, the greater part is laid out in grass plots, intersected by gravel walks. One portion is specially set aside as a playground for the children of the tenants. In the centre is a music pavilion, where a band of eight performers plays twice a week during the summer, at the proprietor's expense. In wet weather the children play under the verandahs, or in the cellars, which are paved. A part of the court is also set apart for the drying of clothes, and on this rows of wooden T-shaped supports have been erected for the tenants' convenience. The buildings are six storeys high, and are almost absolutely fireproof. The houses on the upper storeys are reached by means of outside front balconies, after the fashion common in London. Each flat is thus easily made self-contained, and perfect through ventilation exists. The buildings contain 23 four-roomed tenements, 161 of three rooms, 91 of two rooms, and, in the whole block, only three dwellings of only one room. We do not know if there are any restrictions as to the letting of the flats, but if care were taken to let one-room tenements only to spinsters or widows without children, there would be no objection to a fair sprinkling of such flats in a block. They would be convenient for women, who often would rather live alone than lodge in a strange family, yet whose earnings do not permit of their paying more than a very small rent.

The average size of the two-roomed tenements in this block consists of a living-room (or kitchen), measuring 16 ft. by 10 ft., and a bedroom, the length of which is 16 ft., and the width 7 ft. In addition to this each flat has a scullery and a water-closet. These open from the kitchen, but are in the rear of the building, quite detached, and ventilated directly from the open air. This is the arrangement for the sanitary conveniences throughout the entire block. In the three-room tenements the living-room is of the same size as in the smaller flats, and the bedroom is equally large, while the parlour is 15 ft. long and 8 ft. wide. Another type of three-room flat has the parlour and bedroom of the same size, viz., 16 ft. by 8 ft., while the living-room measures 18 ft. by 10 ft. In the four-room dwellings the living-room is of the same size, but the parlour and bedrooms are somewhat smaller. The ceilings on the first floor are 10 ft. high, but those of the upper storeys only 8 ft. 3 in. Washing is done in the scullery, where tubs are provided, and the drying of clothes on the roof or the courtyard, according as the family lives in one of the three upper or three lower floors. Locked cellars for keeping fuel are also in existence, but the tenants, as a rule, prefer to keep their coals in a box fixed in the kitchen, which holds a quarter of a ton. There are no fireplaces; only slate slabs are placed in the rooms for the tenants' stoves to rest on.

From an English point of view, the rents seem high. A single room and scullery on the ground floor cost 1 dol. 40 cents (5s. 10d.) a week. Two-room tenements run from 1½ to 2½ dols. weekly (6s. 3d. to 10s. 5d.) weekly, according to the height and various advantages of location. The dearest three-room flats rent at 2 dols. 90 cents (12s. 1d.) on the first floor, and diminish 10 cents per floor as you ascend. Four-room dwellings run from 3 dols. 60 cents to 2 dols. 30 cents (15s. to 9s. 7d.) in the same way. A rebate of 10 cents per week has, however, been allowed each May to tenants who have paid their rent promptly in advance during the preceding twelve months. We may thus deduct £1 1s. 8d. annually in calculating the minimum rent. Even so, this leaves £14 as the lowest rent at which one can obtain even a single room in the Riverside Buildings.

These buildings, though in themselves admirable, do not, it seems to us, solve, if, indeed, they touch, the problem of housing the poor. Allowing for the fact that earnings are higher in America than in Europe, we are sure that only the fairly well-to-do among the working-class can pay such rents. The best scheme for filling this gap in New York charities which we have heard of is, it must be admitted, not yet in working order, though it is in progress. It has been evolved from the brains of two young ladies, who have based their plan on solid experience. For a year they lived as working girls in a slum. Thus they found how often the poor have to pay more than the rich for the necessaries of life, simply because of the lack of convenience in their dwellings. In one room they could not see without gas, which meant an expenditure of nearly 30s. a month. They had no place to store coal in, and had to buy it by the bucketful, in which way it cost them about £5 a ton instead of £1. They had no bath and no washtub. In the stifling summer evenings there was no place where they could get a breath of fresh air. Acting on the knowledge thus gained, they have planned a block which shall give all the tenants light, airy rooms, cupboards and coal-bins, hot and cold water, and a bath, which, by inserting a sliding partition, can be transformed into two tubs for the family washing. Balconies will surround the building, and the roof will be made a promenade. They claim that this can all be done at a cost of five per cent. less than such houses usually cost, and they have been successful in getting their plan taken up by a company of well-known people, among whom are Mrs. Josephine Shaw Lowell, a relative of the poet, and Mrs. Pierpoint Morgan, the wife of the banker. Mr. Pierpoint Morgan is the son of Mr. Junius Morgan, the generous benefactor of the National Pension Fund for Nurses; and it is evident that the charities for which the family are famed are to suffer no diminution at his hands.

